# Development of cell-cycle inhibitors for cancer therapy

**DOI:** 10.3747/co.v16i2.428

**Published:** 2009-03

**Authors:** M.A. Dickson, G.K. Schwartz

**Affiliations:** * Department of Medicine, Division of Solid Tumor Oncology, Melanoma and Sarcoma Service, and Laboratory of New Drug Development, Memorial Sloan–Kettering Cancer Center, New York, NY, U.S.A

**Keywords:** Cell cycle, cyclin-dependent kinases, cyclins, phase i clinical trials, phase ii clinical trials

## Abstract

The cell cycle governs the transition from quiescence through cell growth to proliferation. The key parts of the cell cycle machinery are the cyclin-dependent kinases (cdks) and the regulatory proteins called cyclins. The cdks are rational targets for cancer therapy because their expression in cancer cells is often aberrant and their inhibition can induce cell death. Inhibitors of cdks can also block transcription.

Several drugs targeting the cell cycle have entered clinical trials. These agents include flavopiridol, indisulam, AZD5438, SNS-032, bryostatin-1, seliciclib, PD 0332991, and SCH 727965. Phase i studies have demonstrated that these drugs can generally be administered safely. Phase ii studies have shown little single-agent activity in solid tumors, but combination studies with cytotoxic chemotherapy have been more promising. In hematologic malignancies, reports have shown encouraging single-agent and combination activity. Pharmacodynamic studies show that the dose and schedule of these drugs are crucial to permit maximum therapeutic effect.

## 1. INTRODUCTION

With advancing understanding of oncogenesis and apoptosis comes an appreciation of the role cell-cycle regulation plays in malignant transformation. Modulation of the cell cycle also contributes to chemotherapy resistance. The cyclin-dependent kinases (cdks), the essential engines of the cell cycle, are therefore rational therapeutic targets. Over the last several years, a new class of anticancer therapy has been developed and extensively tested: inhibitors of cdks.

These drugs have been tested as single agents with modest results. However, in combination with traditional cytotoxic chemotherapy, they have the potential to overcome drug resistance and to improve cytotoxic efficacy.

## 2. THE CELL CYCLE AND ITS REGULATION

The cell cycle governs the transition from quiescence (G0) to proliferation while ensuring the fidelity of the genetic transcript. The phases associated with dna synthesis (S phase) and mitosis (M phase) are separated by the gaps G1 and G2. The cdks join with regulatory proteins called cyclins to drive the cell through the cycle.

Inhibitory proteins [cdk inhibitors (cdkis)] block specific interactions. The Ink4 (inhibitor of cdk4) class of cdkis (p16^Ink4a^, p15^Ink4b^, p18^Ink4c^, and p19^Ink4^) bind and inhibit cyclin D–associated kinases (cdk2, -4, and -6), and the kinase inhibitor protein (Kip) group of cdkis (p21^Waf1^, p27^Kip1^, and p57^Kip2^) block the cyclin E/cdk2 and cyclin A/cdk2 complexes 1.

The pattern of cyclin expression defines the cell’s progression through the cycle 2,3. At least 9 cdks (cdk1–cdk9) and many cyclins (cyclin A–cyclin T) are known. The cdk/cyclin complexes are activated by specific phosphorylation of the cdk by cdk7/cyclin H, also called cdk-activating kinase 4. Specific complexes regulate each step of the cycle. Cyclins D1–D3/cdk2, -4, and -6 drive progression through G1; cyclin E/cdk2 controls entry into S phase; cyclin A/cdk2 controls S-phase progression; cyclin A/ cdk1 (also known as cdc2) controls G2; and cdk1/ cyclin B facilitates mitosis.

Entry into the cell cycle (G1) is governed by the restriction point, beyond which progression through the cycle is independent of stimuli such as mitogens 5. Mitogens signal through the Ras/Raf/ mapk pathway leading to cyclin D production. The retinoblastoma tumour suppressor gene product (Rb) governs the G1/S transition. In its active state, Rb is hypophosphorylated and inhibits the transcription factors E2F–DP (E2F-1, -2, and -3). Rb is in turn phosphorylated by cyclin D/cdk4/6 and cyclin E/cdk2, modulating its activity 6. When partially phosphorylated, Rb remains bound to E2F-DP, but the transcription factor can still transcribe some genes such as cyclin E. Cyclin E then binds to cdk2, and the complex hyperphosphorylates Rb, releasing the E2F–DP complex and fully activating the E2F transcription factors. S-Phase proteins are then transcribed 7.

Early in S phase, cyclins D and E are degraded 8. Cyclin A/cdk2 governs S-phase progression and the production of proteins involved in dna synthesis 9,10. Cyclin A/cdk2 also inactivates E2F 11–14.

In late S phase and throughout G2, levels of cyclins A and B rise. Cyclin B/cdc2 (cdk1) regulates the S-phase checkpoint. This replication checkpoint monitors progression through S phase and moderates dna synthesis 15,16. It is regulated by the atm (ataxia telangiectasia mutated) and atr (atm and Rad3-related) kinases and Chk1 and Chk2, which prevent cell-cycle progression in the event of dna damage 17,18. These pathways permit a cell to enter mitosis only after successful completion of S phase.

Mitosis is regulated by the anaphase-promoting complex/cyclosome and by degradation of cyclin B 10. The assembly of a bipolar spindle by the centrosome is monitored by a checkpoint that senses microtubule defects or aberrant kinetochore attachment 19–21. Centrosome maturation, regulated by kinases including Polo kinase and Aurora kinase, begins with centriole duplication, which occurs in G1 and is triggered by cyclin E/cdk2 and cyclin D/cdk2 activity. Elongation of the centriole occurs throughout S phase so that by prophase, the cell has two pairs of centrioles 22. Aurora kinase regulates spindle pole structure and duplication and separation of the centriole 23,24. Survivin, regulated by cyclin B1/cdc2, regulates the mitotic spindle and cell viability 25–27.

## 3. THE CELL CYCLE AS A TARGET FOR CANCER THERAPEUTICS

**FIGURE 1:**
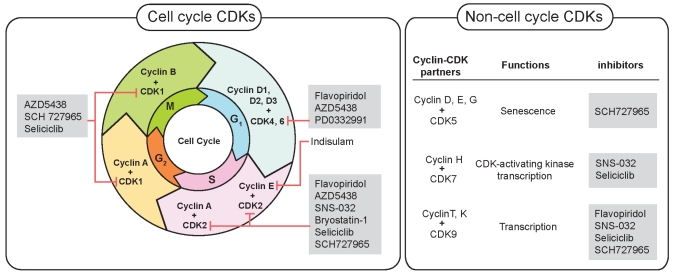
Example of inhibitors that, in early clinical trials, are targeting cyclin-dependent kinases (cdks) acting in or outside of the cell cycle.

The cdks are rational targets for cancer therapy. Their expression is often perturbed in malignancy, and their inhibition can induce apoptosis. Most tumour-suppressor genes and oncogenes are part of pathways that control cellular functions, including cell-cycle entry and exit 28,29. Checkpoint integrity is often lost as a result of inactivation of cdkis or of overexpression of cyclins. For example, loss of p16 function is associated with melanoma, lung, breast, and colorectal tumours 30. Overexpression of cyclin D1 is associated with breast cancer 31,32. Thus, targeting cdks could restore cell-cycle checkpoints and may slow growth or induce apoptosis 33. Figure 1 shows the site of action of cdkis in clinical development.

Inhibitors of cdk also inhibit transcription. A key enzyme in the transcription machinery, rna polymerase ii, is phosphorylated by several cdks 34,35. The most important regulator is cdk9/cyclin T. Inhibition of cdk9/cyclin T by a cdki such as flavopiridol (discussed in the next subsection) leads to inhibition of rna polymerase ii and a decrease in the anti-apoptotic protein Mcl-1 36. Apoptosis is induced.

### 3.1 Flavopiridol

Flavopiridol is a pan–cdk inhibitor that blocks cdk2, -4, and -6 at nanomolar concentrations. *In vitro,* it causes cell-cycle arrest both at the G1/S transition and at the G2/M transition.

Several phase i and phase ii studies of flavopiridol have been reported in a variety of solid tumours and hematologic malignancies. A phase i study in chronic lymphocytic leukemia (cll) noted some encouraging responses. Flavopiridol was administered weekly for 4 of 6 weeks in 52 patients with refractory cll. The patients were treated with a 30–40 mg/m^2^ loading dose followed by 30–50 mg/m^2^ over 4 hours. The dose-limiting toxicity (dlt) was hyperacute tumour lysis syndrome. Partial responses (prs) were achieved in 40% of patients, and those responses were durable, with a median progression-free survival of 12 months 37,38.

Phase ii studies of flavopiridol as a single agent have been completed in metastatic melanoma 39, endometrial adenocarcinoma 40, and multiple myeloma 41. No objective evidence of antitumour activity was observed in the 58 patients treated on those studies. Major toxicities were myelosuppression and diarrhea.

Flavopiridol holds more potential as an enhancer of the effects of cytotoxic chemotherapy. A major phase i study that assessed the combination of flavopiridol and irinotecan enrolled 45 patients. The identified maximum tolerated doses (mtds) were irinotecan 100 mg/m^2^ with flavopiridol 60 mg/m^2^ and irinotecan 125 mg/m^2^ with flavopiridol 50 mg/m^2^. Partial responses were observed in 3 patients 42.

Further laboratory work defined the mechanism of activity. By inhibiting cdk9, flavopiridol inhibited Rad51, a dna repair protein involved in homologous recombination. This protein sensitizes cells, in a p53-dependent manner, to induction of apoptosis by topoisomerase i poisons 43.

Other combinations of flavopiridol with chemotherapy have also shown modest activity. A phase i study of flavopiridol with carboplatin and paclitaxel was performed in 18 patients with previously-untreated non-small-cell lung cancer (nsclc). Adverse events included nausea, asthenia, and diarrhea. The mtd of flavopiridol was 70 mg/m^2^ with paclitaxel 175 mg/m^2^ and carboplatin auc (area under the curve) 5. Of 12 evaluable patients, 8 achieved a pr 44.

Two phase i studies of flavopiridol in combination with docetaxel have been reported. In the first, 10 patients were treated with flavopiridol and docetaxel given once every 21 days. The dlts were neutropenia and infection. The mtd was docetaxel 60 mg/m^2^ followed 24 hours later by flavopiridol 50 mg/m^2^ over 24 hours 45. In the second study, both drugs were administered weekly for 3 in 4 weeks in 27 patients with advanced solid tumours. The mtd was docetaxel 35 mg/m^2^ followed 4 hours later by flavopiridol 70 mg/m^2^. The best response was an extraordinary complete response in pancreatic cancer. Four prs were observed in various tumours 46.

A phase i study of flavopiridol in combination with either cisplatin or carboplatin in 39 patients has been reported. The mtd was 60 mg/m^2^ cisplatin and 100 mg/m^2^ flavopiridol over 24 hours. Carboplatin auc 2 with 100 mg/m^2^ flavopiridol over 24 hours was deemed intolerable because of significant toxicity, including fatigue, nausea, diarrhea, and myelosuppression. The best response was stable disease (sd) 47.

In a phase ii study, flavopiridol 50 mg/m^2^ over 1 hour 3 times daily, in combination with cytarabine and mitoxantrone in 49 patients with poor-risk acute myelogenous leukemia (aml), showed encouraging activity. Tumour lysis occurred in more than half the patients. Complete responses were observed in 75% of patients who were either previously untreated or who had experienced early relapse 48.

In additional to the clinical activity in cll and aml already described, preclinical activity of flavopiridol has also been observed in acute lymphoblastic leukemia 49. A recently developed liposomal formulation of the drug ought to increase the drug’s half-life, its auc, and perhaps its efficacy 50.

### 3.2 Indisulam

Indisulam (E7070) is a synthetic sulphonamide that targets the G1 phase of the cell cycle by depleting cyclin E, inducing p53 and p21, and inhibiting cdc2 phosphorylation 51.

A phase ii study demonstrated *in vivo* pharmacodynamic (pd) activity: post-treatment biopsies showed a decrease in Rb phosphorylation. The short duration of the pd effect led to the conclusion that continuous dosing would likely be required. This finding highlighted the importance of the dose schedule in maintaining a cytostatic effect of drugs that target the cell cycle 52.

Other notable single-agent studies include a phase ii trial in malignant melanoma. The 28 patients enrolled were treated at a dose of 700 mg/m^2^ every 3 weeks. No objective responses were observed, but minor responses and sd were seen 53.

In a phase ii study in second-line therapy for nsclc, patients were randomized to receive indisulam every 3 weeks either as a single intravenous (IV) dose of 700 mg/m^2^ on day 1 or 130 mg/m^2^ IV on days 1–5. In the 44 patients treated, only minor responses were seen. However, evidence of pd targeting was observed: flow cytometric analysis of endobronchial and metastatic disease revealed a reduction in the fraction of cycling cells and an increase in apoptosis following indisulam as compared with pretreatment levels. Nevertheless, the drug was considered to have no significant single-agent activity 54.

Combination studies with chemotherapy have also been pursued. A phase i study of indisulam with carboplatin found the mtd to be indisulam 500 mg/m^2^ on day 1 with carboplatin auc 6 given every 4 weeks. Toxicities were thrombocytopenia and neutropenia, and significant myelosuppression prevented treatment on the originally-planned 3-week cycle. The best response was sd 55.

In a phase ii study of indisulam in combination with capecitabine, 35 patients were treated. The mtd for multiple treatment cycles was indisulam 500 mg/ m^2^ on day 1 and capecitabine 1250 mg/m^2^ twice daily on days 1–14 of each 21-day cycle. The best response was 2 prs. Toxicities included myelosuppression, stomatitis, and hand–foot syndrome 56.

### 3.3 AZD5438

AZD5438 is a novel cyclin-dependent kinase inhibitor with preclinical activity against a range of human tumour xenografts. In a phase i study in healthy volunteers, the drug was found to have a relatively short half-life of 1–3 hours 57,58. Nevertheless, pd effects were demon-strated; the drug led to statistically significant reductions in the ratio phospho-pRb /total pRb detected at 1.5 hours post-dose, but the effect disappeared at 6 hours post-dose. Thus, given the short half-life and close pharmacokinetic–pharmacodynamic relationship, a sustained-release formulation or multiple daily dosing will be required for further drug development.

A second phase i study of AZD5438 in patients with advanced solid malignancies has recently been completed. Results have yet to be reported (search for “NCT00088790” at www.clinicaltrials.gov/ct2/search).

### 3.4 SNS-032 (BMS-387032)

SNS-032 is a potent and selective inhibitor of cdk2, -7, and -9. A phase i study of the drug in patients with metastatic solid tumours was recently published. The drug was administered as a weekly 1-hour infusion. Toxicities included fatigue and nausea. No dlt was observed. Some patients received an oral solution for one of the doses, and pharmacokinetic studies demonstrated that oral administration may be feasible 59.

A second phase i study of SNS-032 in advanced B-cell lymphoid malignancies is ongoing (search for “NCT00446342” at www.clinicaltrials.gov/ct2/search).

### 3.5 Bryostatin-1

Bryostatin-1 is a macrocyclic lactone that modulates the cell cycle, inducing p21 and inactivating cdk2 60. In a phase i trial, the drug showed limited single-agent activity in melanoma, ovarian cancer, and non-Hodgkin lymphoma 61. Bryostatin has been evaluated in combination with chemotherapy in a number of phase i and phase ii studies.

A phase i trial of bryostatin and gemcitabine was conducted in 36 patients with advanced solid tumours. Gemcitabine was administered IV over 30 minutes and was followed by bryostatin IV over 24 hours on days 1, 8, and 15 of a 28-day cycle. Common toxicities were anemia, neutropenia, and thrombocytopenia. The best response was sd in 8 patients. The recommended phase ii dose was bryostatin 35 μg/m^2^ and gemcitabine 1000 mg/m^2 62^.

Another phase i study assessed bryostatin and fludarabine in patients with cll or indolent lymphoma. Fludarabine was given daily for 5 days, and a single dose of bryostatin was given by a 24-hour continuous infusion either before or after the fludarabine. The study concluded that bryostatin can be administered safely and tolerably with full-dose fludarabine (25 mg/m^2^ daily for 5 days). The recommended bryostatin phase ii dose is 50 μg/m^2^ for both sequences. The combination showed moderate activity, and responses were seen in patients who had previously been treated with fludarabine 63.

A phase ii study of bryostatin and paclitaxel was performed in patients with gastric or gastroesophageal junction adenocarcinoma. Paclitaxel 80 mg/m^2^ IV over 2 hours was given on day 1, with bryostatin 40 μg/m^2^ IV over 1 hour on day 2 each week for 3 consecutive weeks in 4. There were 35 evaluable patients. The confirmed pr rate was 29%. Grade 3 cumulative myalgias occurred in 55% of patients 64.

Another phase ii study assessed bryostatin and paclitaxel in advanced esophageal cancer. The initial dose was paclitaxel 90 mg/m^2^ on day 1 and bryostatin 50 μg/m^2^ on day 2 weekly for 3 consecutive weeks in 4. In 22 evaluable patients, the pr rate was 27%. Grades 3 and 4 myalgias requiring dose reduction were seen in 50% of patients. The trial was closed early because of toxicity; thus, although antitumour activity was observed, further development will not be pursued 65.

### 3.6 Seliciclib

The agent seliciclib [CYC202, (*R*)-roscovitine] is a potent oral inhibitor of cdk2/cyclin E, cdk1/cyclin B, cdk7/cyclin H, and cdk9/cyclin T1 66,67. Seliciclib suppresses genes that inhibit apoptosis and has single-agent *in vitro* activity against a range of tumours 68–71. *In vivo* activity has also been reported for seliciclib against human colon and uterine cancer xenografts 72.

A phase i study of seliciclib in 22 patients has been completed in Europe 73. The mtd was 800 mg twice daily given for 7 in every 21 days. Common side effects were nausea, lethargy, and anorexia. The dlts were hypokalemia, rash, and fatigue. No objective responses were reported, but disease stabilization occurred in 8 patients and lasted 18 weeks in a patient with ovarian cancer.

A phase i study of seliciclib in combination with cisplatin and gemcitabine was performed in the first-line treatment of 27 patients with nsclc. Seliciclib was administered for 4 in every 7 days. The dlts consisted of liver enzyme elevation, nausea, vomiting, and transient hypokalemia. The mtd was seliciclib 800 mg twice daily with gemcitabine 1000 mg/m^2^ and cisplatin 75 mg/m^2^ Among 14 evaluable patients, 6 prs were observed^74^

A phase ii study of seliciclib as a single agent in patients with previously-treated nsclc has been closed. No data have yet been reported (search for “NCT00372073” at www.clinicaltrials.gov/ct2/search).

### 3.7 PD 0332991

PD 0332991, a pyrido[2,3-δ]pyrimidine-7-one, is a selective inhibitor of cdk4 and cdk6^75^. In low micromolar concentrations in *in vitro* and xenograft models, it inhibited a panel of Rb-positive solid tumour cell lines^76^,^77^. The drug was also tested *in vitro* against mantle cell lymphoma (mcl)^78^. Translocation-mediated constitutive expression of cyclin D, the partner of cdk4 and -6, is typical of mcl. As predicted, cells are sensitive to PD 0332991 at low-nanomolar concentrations.

A phase i clinical trial with PD 0332991 in patients with Rb-positive advanced solid tumours was performed. The principal and dose-limiting toxicity of PD 0332991 is myelosuppression. The mtd is 125 mg daily for 21 in every 28 days. On a shorter schedule, slightly higher doses were tolerated^79^.

A trial of PD 0332991 in mcl is ongoing (search for “NCT00420056” at www.clinicaltrials.gov/ct2/search), as are combination studies with letrozole for breast cancer (search for “NCT00721409”) and with bortezomib and dexamethasone for multiple myeloma (search for “NCT00555906”).

### 3.8 SCH 727965

SCH 727965 is a novel pyrazolo[1,5-α]pyrimidine that potently and selectively inhibits cdk1, cdk2, cdk5, and cdk9. It induces apoptosis in tumour cell lines and growth inhibition or regression in xenograft models. A phase i study is underway with interim results in 23 patients reported. The drug is administered by 2-hour IV infusion once every 21 days. The most common and dose-limiting toxicity is neutropenia. No objective responses were observed. The drug was safe and well tolerated below the maximum administered dose of 58 mg/m^2 80^.

A randomized phase ii study comparing SCH 727965 with erlotinib in patients with nsclc and comparing SCH 727965 with capecitabine in patients with advanced breast cancer is underway (search for “NCT00732810” at www.clinicaltrials.gov/ct2/search). A second phase ii study in acute leukemia is planned (search for “NCT00798213”).

## 4. CONCLUSIONS

Phase i studies have demonstrated that cdkis can be safely administered to patients with advanced cancer. Doses with demonstrable pd effects can be achieved. Single-agent activity in solid tumours has, in general, been disappointing. However, in hematologic malignancies, which may be more sensitive to blockade of cell cycling and induction of apoptosis, encouraging activity has been observed. Examples include flavopiridol as a single agent in cll or in combination with cytarabine and anthracycline in aml. The cdkis may also contribute to overcoming drug resistance, as in the case of flavopiridol combined with fludarabine in fludarabine-refractory cll.

Activity of these agents in solid tumours has been more modest, and the evidence argues that combination studies with other agents should be pursued, but expectations for response should be modest. Drugs that arrest the cell cycle may, at best, result in stabilization of disease. Nevertheless, the preclinical evidence of induction of apoptosis suggests that cell-cycle inhibitors, if given on the right schedule with the right combination of drugs, may cause tumours to regress.
